# Trapping Conformational States Along Ligand-Binding Dynamics of Peptide Deformylase: The Impact of Induced Fit on Enzyme Catalysis

**DOI:** 10.1371/journal.pbio.1001066

**Published:** 2011-05-24

**Authors:** Sonia Fieulaine, Adrien Boularot, Isabelle Artaud, Michel Desmadril, Frédéric Dardel, Thierry Meinnel, Carmela Giglione

**Affiliations:** 1 CNRS, ISV, UPR2355, Gif-sur-Yvette, France; 2 Université Paris Descartes, UMR8601, Paris, France; 3 CNRS, UMR8601, Paris, France; 4 Université Paris-Sud, IBBMC, UMR8619, Orsay, France; 5 CNRS, IBBMC, UMR8619, Orsay, France; 6 Université Paris Descartes, UMR8015, Paris, France; 7 CNRS, UMR8015, Paris, France; Brandeis University, United States of America

## Abstract

For several decades, molecular recognition has been considered one of the most fundamental processes in biochemistry. For enzymes, substrate binding is often coupled to conformational changes that alter the local environment of the active site to align the reactive groups for efficient catalysis and to reach the transition state. Adaptive substrate recognition is a well-known concept; however, it has been poorly characterized at a structural level because of its dynamic nature. Here, we provide a detailed mechanism for an induced-fit process at atomic resolution. We take advantage of a slow, tight binding inhibitor-enzyme system, actinonin-peptide deformylase. Crystal structures of the initial open state and final closed state were solved, as well as those of several intermediate mimics captured during the process. Ligand-induced reshaping of a hydrophobic pocket drives closure of the active site, which is finally “zipped up” by additional binding interactions. Together with biochemical analyses, these data allow a coherent reconstruction of the sequence of events leading from the encounter complex to the key-lock binding state of the enzyme. A “movie” that reconstructs this entire process can be further extrapolated to catalysis.

## Author Summary

The notion of induced fit when a protein binds its ligand—like a glove adapting to the shape of a hand—is a central concept of structural biochemistry introduced over 50 years ago. A detailed molecular demonstration of this phenomenon has eluded biochemists, however, largely due to the difficulty of capturing the steps of this very transient process: the “conformational change.” In this study, we were able to see this process by using X-ray diffraction to determine more than 10 distinct structures adopted by a single enzyme when it binds a ligand. To do this, we took advantage of the “slow, tight-binding” of a potent inhibitor to its specific target enzyme to trap intermediates in the binding process, which allowed us to monitor the action of an enzyme in real-time at atomic resolution. We showed the kinetics of the conformational change from an initial open state, including the encounter complex, to the final closed state of the enzyme. From these data and other biochemical and biophysical analyses, we make a coherent causal reconstruction of the sequence of events leading to inhibition of the enzyme's activity. We also generated a movie that reconstructs the sequence of events during the encounter. Our data provide new insights into how enzymes achieve a catalytically competent conformation in which the reactive groups are brought into close proximity, resulting in catalysis.

## Introduction

Flexibility of proteins around their active site is a central feature of molecular biochemistry [1]–[5]. Although this has been a central concept in biochemistry for half a century, the detailed mechanisms describing how the active enzyme conformation is achieved have remained largely elusive, as a consequence of their transient nature. Direct structural evidence and/or kinetic analyses have only recently emerged [6]–[10]. Three classic “textbook” models are used to describe the formation of the ligand-enzyme complex: (i) the Fischer's “lock-and key” model, (ii) the Koshland's induced-fit model, and (iii) the selected-shift model or conformational selection mechanism [6]–[8],[11]–[13]. In the Fischer's “lock-and key” model, the conformations of free and ligand-bound proteins are essentially the same. In the induced-fit model, ligand binding induces a conformational change in the protein, leading to the precise orientation of the catalytic groups and implying the existence of initial molecular matches that provide sufficient affinity prior to conformational adaptation [14]. In contrast, the selected-fit model assumes an equilibrium between multiple conformational states, in which the ligand is able to select and stabilize a complementary protein conformation. In this case, the conformational change precedes ligand binding, in contrast to the induced-fit model in which binding occurs first. The conformational selection and/or induced-fit processes have been shown to be involved in a number of enzymes [12],[13],[15],[16]. For several of these studies, conformational selection is proposed because the experimental data support that, even in the absence of the ligand, the enzyme samples multiple conformational states, including the ligand-bound (active) state [6]. Although direct structural evidence and/or kinetic analyses have provided clues [6]–[8],[12],[13],[16], how we can distinguish whether a protein binds its ligand in an induced- or selected-fit mechanism remains critical and often controversial.

**Figure 1:**
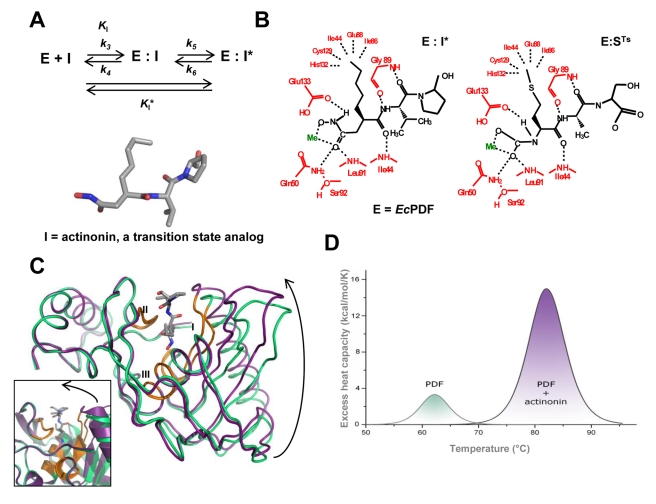
Slow, tight-binding inhibition of PDF by actinonin induces conformational change in the protein. (A) Inhibition by a two-step mechanism, involving a tightening of the initial enzyme-inhibitor complex (E·I) to form a more stable complex (E·I*), with the chemical structure of actinonin (I), the natural inhibitor of PDF enzymes (E). (B) Structures of *Ec*PDF bound to actinonin (left) and to the transition state resulting from the cleavage of its substrate, *Fo*-Met-Ala-Ser (right) [34],[35]. (C) Superimposition of free and actinonin-bound *At*PDF indicated in green and purple, respectively. The three conserved motifs of the PDF enzymes family are indicated in orange and numbered I, II, and III. Molecules A of both models were superimposed, resulting in an r.m.s.d. of 0.9 Å for 100% of the C*α*. Left inset, close-up comparison of the open and closed forms figured in the ribbon representation. (D) Baseline-corrected DSC thermograms of free and actinonin-bound WT *At*PDF recorded under the same experimental conditions.

The enzyme-inhibitor interaction is a form of molecular recognition that is more amenable to investigation than the enzyme-substrate interaction as there is no chemical transformation of the ligand during this process. In this context, slow, tight-binding inhibition is an interesting interaction process, as it closely mimics the substrate recognition process and has been shown to be commonly involved in adaptive conformational changes [12],[17],[18]. In slow, tight-binding inhibition, the degree of inhibition at a fixed concentration of compound varies over time, leading to a curvature of the reaction progress curve over time during which the uninhibited reaction progress curve is linear [19]. Indeed, the slow, tight-binding inhibition is a two-step mechanism that depends on the rate and strength of inhibitor interactions with the enzyme. Binding of the inhibitor (I) to the enzyme (E) leads to the rapid formation of a non-covalent enzyme-inhibitor complex (E:I) followed by monomolecular slower step (*k*_5_) in which the E:I is transformed into a more stable complex (E:I*) that relaxes and dissociates at a very slow rate, mainly inferred by the *k*_6_ value when *k*_6_<<*k_5_*<<*k_4_*, (Figure 1A; see also footnote f in Table 1).

**Table 1:** Comparison of the main kinetic and thermodynamic parameters describing the inhibition of PDF by actinonin.

Parameter	*At*PDF^a^	*Ec*PDF^a^	*Bs*PDF2^a^^,^ b
*K*_I_ (nM)^d^	140±10	112±10	185±15
*K*_I*_ (nM)^c^	0.9±0.5	1.3±0.2	2.9±0.8
*K*_I_/*K*_I*_	155±15	86±10	64±7
*k*_5_ (s^−1^) ×10^3d^	63±9	170±20	72±8
*k*_6_ (s^−1^) ×10^4d^	4±1	19±2	11±3
*k_4_* (s^−1^)^e^	140±10	112±10	185±15
*t_1/2_* (min)^f^	29±5	6±1	1.1±0.2

a The enzyme concentrations used in the assay were 100, 50, and 25 nM for *At*PDF, *Ec*PDF, and *Bs*PDF2, respectively.

b Data from [49].

c Prior to kinetic analysis for determination of the *K*_I_* value, actinonin was incubated at the final concentration in the presence of the studied enzyme set for 10 min at 37°C. The kinetic assay was initiated by the addition of a small volume of the substrate.

d For determination of *K*_I_, *k*_5_, and *k*_6_ values, actinonin was not preincubated with the enzyme. The kinetic assay was initiated by the addition of the enzyme.

e *k_4_* corresponds to the kinetic constant of the dissociation of the primary enzyme-actinonin complex. It is assumed that the rate of complex association is diffusion-limited (see Table 7.3 in [19]), that is, *k_3_*—the kinetic constant of the association of the primary enzyme-actinonin complex—is 10^9 M−1^.s^−1^.

f *t_1/2_* is 0.693(*k_4_*+*k_5_*+*k_6_*)/*k_4_k_6_* (see case of induced fit and calculation in Table 1 of [12]). In this case, *t_1/2_*∼0.693/*k_6_* because *k_6_*<<*k_5_*<<*k_4_*.

Although only a few studies have investigated the mechanisms of slow, tight-binding inhibitors, such molecules are favored for use as therapeutics, as they usually exhibit unique inhibitory properties, including selective potency and long-lasting effects [20]–[26]. Here, we explore the precise structural inhibitory mechanism of actinonin (Figure 1A; [27]), which is a slow, tight-binding inhibitor of peptide deformylase (PDF), a metal cation-dependent enzyme [28],[29]. The function of the active-site metal is to activate the reactive water molecule involved in peptide hydrolysis [30]. PDF is the first enzyme in the N-terminal methionine excision pathway, an essential and ubiquitous process that contributes to the diversity of N-terminal amino acids [31],[32]. Actinonin is a natural product with antibiotic activity that inhibits PDF by mimicking the structure of its natural substrates (nascent peptide chains starting with *Fo*-Met-Aaa, where *Fo* is a formyl group and Aaa is any amino acid) in their transition state (Figure 1B). The transition state inhibitor actinonin, as well as other structurally related inhibitors, has been shown to systematically exhibit a “slow-binding” inhibition behavior (Figure 1A), regardless of the organism of origin of the PDF [29],[33].

Using structural, biocomputing, and enzymatic analyses, we were able to (i) reveal that the free enzyme is in an open conformation and that actinonin induces transition of the enzyme into a closed conformation; (ii) show that there is no evidence for the occurrence of a closed conformation in the apostructure of the open enzyme, which, together with detailed kinetic analyses, makes the closed form fully compatible with an induced-fit model; and (iii) identify the sequence of molecular events leading to the final, bound, closed complex (E:I*). Moreover, using several rationally designed point mutants of the enzyme, ligand-induced intermediates, which mimic conformational states that normally would not be expected to accumulate with the wild-type (WT) enzyme, were trapped. These conformations recapitulate physical states that the WT enzyme must pass through during its overall transition from the apo-enzyme to the E:I* complex. “Freezing” of ligand-induced intermediate states provides direct evidence for an induced-fit mechanism and allows the reconstruction of a virtual “movie” that recapitulates this mechanism. Since PDF is one example of an enzyme remaining active in the crystalline state and because actinonin closely mimics the natural substrates bound to PDF in the transition state as shown previously with the *Escherichia coli* form (*Ec*PDF; see Figure 1B) [34],[35], we propose a model suggesting that induced fit also contributes to efficient catalysis.

## Results

### Slow, Tight Binding of the Transition-State Analog Actinonin to Peptide Deformylase

In the present study, at the atomic level we explored the precise inhibitory mechanism of actinonin on *Arabidopsis thaliana* PDF1B (*At*PDF), a close eukaryotic homologue of *Ec*PDF (Figure S1) [36],[37]. Measurements of the kinetic parameters of the second step of the binding mechanism (*k*_5_) revealed a timescale in the 10-s range (Table 1), which is consistent with the collective motion of a large domain [4],[5]. This finding is supported by NMR studies [38],[39], which showed that actinonin binding induces drastic changes in the heteronuclear single quantum coherence (HSQC) spectrum of *Ec*PDF, since most resonances undergo significant shifts that affect a large part of the structure [40],[41]. The existence of alternative conformational states of *Ec*PDF is further supported by recent biophysical studies [42]. Previously reported snapshots of a series of different conformations of the enlarged and mobile loop—the so-called CD loop—of the dimeric PDF from *Leptospira interrogans* PDF (*Li*PDF) in the presence or absence of inhibitor led to the hypothesis of the existence of an equilibrium between a closed and open form of the CD-loop of PDF enzymes, suggesting a selected-shift model to the authors [43]. Taken together, these data suggest that the binding of actinonin to PDF is accompanied or preceded by conformational changes within the enzyme. Paradoxically, this proposal has not been currently supported by the available structural data. Indeed, free and complexed crystal structures have provided no evidence for any significant conformational change in PDF structure induced by the binding of ligand [35],[43]–[47].

Tight inhibition in the closed state is associated with the *K*_I_* apparent equilibrium constant (Figure 1A). A *K*_I_* value (see Table 1 and Materials and Methods for the biochemical definition of *K*_I_*) of 0.9 nM for actinonin could be measured for *At*PDF; that is, a value very similar to that obtained for bacterial PDFs, including *Ec*PDF and *Bacillus stearothermophilus* PDF2 (*Bs*PDF2, Table 1). Tightening of the initial encounter complex (E:I) resulted in a final complex (E:I*) in which the potency of actinonin (*K*_I_/*K*_I*_) was enhanced by more than two orders of magnitude and exhibited a very slow off-rate (*k_6_*, Table 1). The dissociation constant value of *At*PDF for actinonin was also assessed using isothermal titration calorimetry (ITC) experiments (Table S1 and Figure S2A). The corresponding ITC titration curves (Figure S2A) are consistent with a very strong affinity of the ligand for the enzyme [48], enabling us to determine an accurate *K_d_*. Moreover, these studies generated values similar to those measured by other means for *At*PDF and *Ec*PDF [42],[49].

### Ligand-Induced Conformational Closure of *At*PDF in the Crystalline State

Occurrence of a conformational change induced by drug binding was visualized via the resolution of several crystal structure forms of *At*PDF, the free form and/or in a complex with actinonin (Table S2). The data reveal a structural switch between the two forms that can account for both the thermodynamic and kinetic data. The enzyme was observed in two states, a novel open apo-form and a closed, induced, actinonin-bound complex (Figure 1C). Binding of actinonin resulted in a tightening of the active site through the collective closure of the entire N-terminal portion of the protein (strands *β*1, *β*2, and *β*3; helix α1; and CD-loop, see Movies S1 and S2, Figure 1C, and Figure S1). The amplitude of the structural change was maximal for Pro60 (Figure S1), the Cα of which was shifted 4 Å upon actinonin binding. This collective movement involved the formation of a “super β-sheet” as the result of the large rearrangement of β-strands 4 and 5 relative to the rest of the structure in which actinonin forms an additional strand bridging the two β-sheets (*β*1 and*β*2) on either side of the active site (Figure 1D and Figure S1B). As actinonin is a peptide-like compound (see Introduction and Figure 1B), this behavior closely mimics what occurs in the natural protein substrates of PDF, which also form this strand-bridging interaction. This phenomenon also accounts for the strong stabilization of the protein by actinonin, which was also challenged by differential scanning calorimetry (DSC) experiments: the *T*_m_ of *At*PDF increased from 61°C to 81°C upon binding of the inhibitor (Figure 1D, see also below).

**Figure 2:**
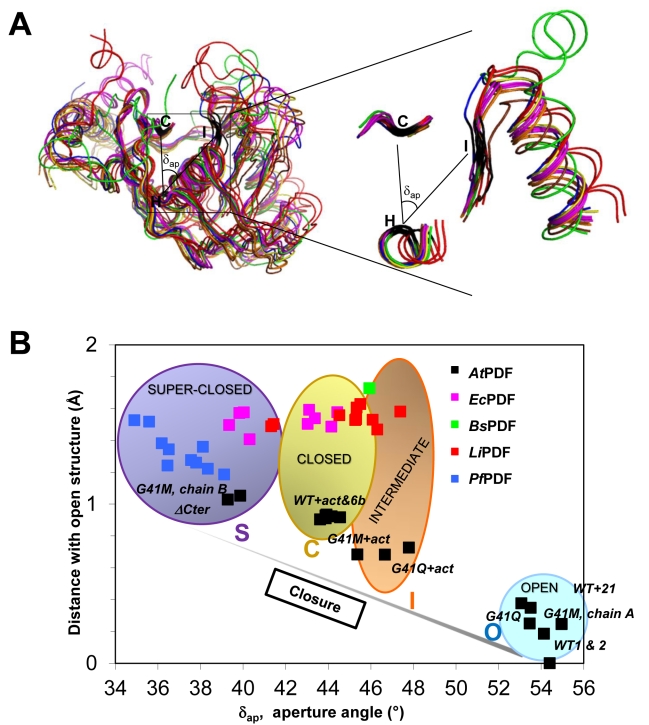
Four distinct conformational states of PDF enzymes. (A) *At*PDF and several other representative bacterial PDFs are superimposed. A zoom is displayed on the right of the panel. Superimpositions were realized using “module superpose” in the CCP4i package and the “secondary structure matching” tool. The extent of aperture/closure of PDF enzymes was assessed primarily by the measurement of the aperture angle (δ_ap_), the angle made between the Cα of three strictly conserved residues (C, H, and I) of all PDFs, each characterizing a secondary crucial structure module of the active site crevice, namely *β*_4_, *α*_2_, and *β*1 (see Figure S1C). Each single residue belongs to one of the three conserved motifs (motifs 2, 3, and 1, respectively) and corresponds respectively to Cys91, His137, and Ile42 in *At*PDF. The δ_ap_ was measured in each case (see B). (B) The δ_ap_ values combined with those of the r.m.s.d. associated with the superimposition of the open structure of *At*PDF allows the identification of four conformational states: open (O), intermediate (I), closed (C), and super-closed (S). We compared *At*PDF1B (this work and PDB CODE 3CPM; brown, orange, and yellow in A and B; black in C), *Ec*PDF (1BS7, free enzyme; 1BS6, with Met-Ala-Ser; 1G2A, with actinonin; magenta), *Bs*PDF2 (1LQY, with actinonin; green), *Li*PDF (1SV2, free; 1SZZ, with actinonin; red), and *Pf*PDF (1JYM, free; blue).

Thus far, this closure of the enzyme induced by actinonin is part of the rare structural evidence for the slow, tight-binding mechanism at an atomic scale. The open state, which has never been observed, was captured not only in the two molecules of the asymmetric subunit but also in different crystals and under two distinct crystallization conditions (Table S2 and Figure 2). All r.m.s.d. values were smaller than 0.25 Å. The closure is very unlikely to result from crystal packing constraints, as soaking the apo-*At*PDF crystals in a solution containing actinonin induced the structural transition from the open to the closed state within the crystals without cracking them or altering their diffracting power. Thus, crystal packing is compatible with both states of the enzyme (Figure S3). Therefore, the open structure most likely corresponds to a stable state in solution.

**Figure 3:**
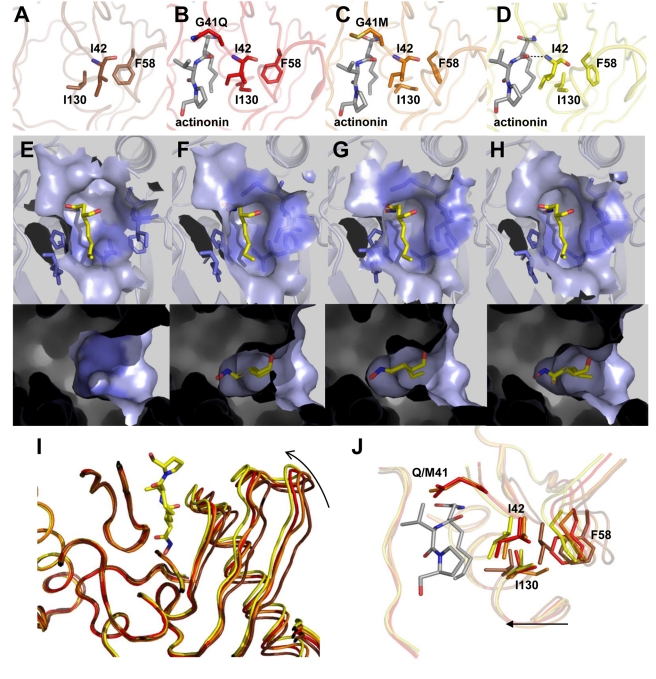
Effect of actinonin binding on the conformation of key residues in PDF. Conformation of key residues Ile42, Phe58, and Ile130 in the different complexes: (A) in unbound WT *At*PDF, (B and C) in the structure of G41Q and G41M actinonin-bound variants, respectively, and (D) of actinonin-bound WT protein. In the final complex (D), a hydrogen bond is formed between actinonin and the peptidic bond, which links Gly41 and Ile42. During the deformylation reaction, which is catalyzed by the PDF enzyme, the N-terminal formyl-methionine fits into the S1' pocket. The solvent-accessible surface of this pocket is represented here, and only the aliphatic chain of actinonin is shown, mimicking the N-terminal methionine. (E) Free WT enzyme with the S1' pocket shown open in two orientations (top and bottom). (F and G) S1' pocket in the G41Q and G41M variant structures, respectively, shown in two orientations (top and bottom). (H) After the complete conformational modifications of actinonin-bound WT protein induced by actinonin binding, the S1' pocket is shown closed in two orientations (top and bottom). (I) The four models are superimposed; the ligand-binding site is magnified: unbound WT *At*PDF; G41Q and G41M actinonin-bound enzyme; and WT actinonin-bound enzyme are indicated in brown, red, orange, and yellow, respectively. Actinonin is indicated by lines. (J) A detailed view of the *At*PDF ligand-binding site for all the complexes, which are superimposed, as indicated in the same colors. Arrows indicate the direction of the closing movement within the enzyme, from the open, unbound state to the closed, bound state.

The closed final conformation was identical to that previously reported for PDF complexes obtained either with actinonin or with a product of the reaction [34],[35],[44],[50], indicating that this structure is common for the ligands (compare Figures 1B and 2A, and Figure S4). Hydrogen bonding was also conserved, especially the bond between the backbone nitrogen of Ile42 (corresponding to Ile44 in *Ec*PDF, see Figure 1B and Figure S5A) and the alkyl carbonyl chain of actinonin, which potently contributes to the formation of the super β-sheet (Movie S2 and Figure S1B, see also below). Between the open and closed states, the side chains of Ile42, Phe58, and Ile130 underwent significant structural changes (Figure 3A and D and Figure S6), corresponding to a hydophobic pocket rearrangement, with Ile42 being the most affected (Figure 3). Interestingly, Ile42 is the second residue of the conserved active-site motif G_41_IGLAAXG (motif 1) that was previously shown to be essential for activity [51].

To assess and visualize the differences between the two states, two independent structural parameters were measured: the r.m.s.d. value with respect to the open form and the aperture angle (δ_ap_), which measures the angle made between the N- and C-domains through three fixed-points, corresponding to the Cα of three conserved residues, each sitting in one of the three conserved motifs (Figure 2A). The bi-dimensional graph of these two parameters is a good representation of the closing motion snapshots (Figure 2B) shown in Movie S1. With this tool at this stage, two states could be defined: the closed (C) and open (O) states (Figure 2B).

### Evidence for a Pure Induced-Fit Mechanism in the Binding of Actinonin to *At*PDF

Recent quantitative analyses of both conformational selection and induced fit have led to an integrated continuum—a so-called “flux-description”—of these two limiting mechanisms [16]. According to this model, conformation selection tends to be preferred at low ligand concentrations (µM range)—that is, using detailed kinetic studies—whereas induced fit dominates at high ligand and enzyme concentrations (mM range) obtained, for instance, in NMR or crystallographic approaches. Structural studies are most useful to reveal subpopulations of biological significance.

**Figure 4:**
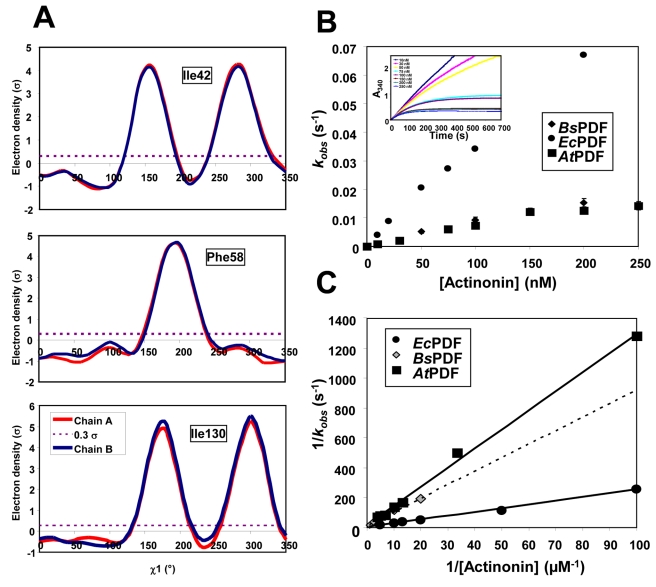
Evidence for an induced fit in crystalline and solution states of *At*PDF. (A) Absence of evidence for alternative conformers in the crystalline state of *At*PDF. Ringer plots of electron density (r) versus χ^1^ angle for representative residues of the 3D apo-structure of *At*PDF. Data were obtained with the 3M6O, 3PN2, and 3PN3 datasets (2.0 and 1.3 Å resolution, respectively, see Table S1). The secondary peaks in the Ile residues are observed because Ile is a branched amino acid. To reveal an alternative conformation with Ile, three peaks should be observed. (B) *k_ob_*_s_ is a saturable function of actinonin with various PDFs, including *At*PDF. Data obtained for *k_ob_*_s_, the experimentally observed pseudo-first-order rate constant for the approach to equilibrium between the free components and the binary PDF-actinonin complex, were obtained at various concentrations of actinonin in the presence of *Ec*PDF, *At*PDF, and *Bs*PDF2. A direct plot is shown. *Inset*, time-course measurement of deformylation as a function of varying actinonin concentrations. (C) Inverted plot of the data in panel B, which is expected to be a straight line if the *k_obs_* is >>*k_6_* in the case of induced fit [19]. The correlation coefficient of each line is 1.00, 0.99, and 1.00 for *At*PDF, *Bs*PDF2, and *Ec*PDF, respectively, indicative of the accuracy of the conclusion.

We investigated the existence of lowly populated, alternative conformations of apoPDF. To probe the occurrence of alternate conformers in the crystalline state of PDF, the new Ringer program is the most suitable investigation tool [52],[53]. Ringer searches for evidence of alternate rotamers by systematically sampling electron density maps—free of model bias—around the dihedral angles of protein side chains. Two independent WT open datasets of the apoenzyme, including a high-resolution set (1.3 Å), were used in the analysis. Ringer analysis revealed the existence of only one rotamer of most side chains of either molecule in the asymmetric unit, including the three main residues primarily involved in conformation change—that is, Ile 42, Phe58, and Ile130 (Figure 4A). Ringer analysis showed evidence for unmodeled alternate conformers for very few residues, including Ile121 and Phe87, or Phe119 to a much lesser extent (Figure S7). There is therefore no evidence for the occurrence of a closed conformation in the apostructure of *At*PDF, supporting the hypothesis that the conformational change was essentially induced by the binding of actinonin rather than from conformational selection among multiple states occurring in the crystalline state.

To further investigate the mechanism involved, we followed a kinetic approach aimed at discriminating between induced fit and population shift at low ligand concentrations (sub-µM range) [12]. The experimentally observed pseudo-first-order rate constant for the approach to equilibrium between the free components and the binary *At*PDF-actinonin complex (*k_obs_*) was measured and plotted as a function of actinonin concentration. This plot yielded a hyperbolic saturation curve with a positive slope, as fully expected for a pure induced-fit mechanism (Figure 4B and C). In contrast, if the enzyme sampled two or more conformational states, the curve would imply that the value of *k_obs_* decreases with increasing ligand concentration (see, for instance, curve C in Figure 1 in [12]). The same conclusion can be reached for *Ec*PDF and *Bs*PDF2 (Figure 4B and C) and was already reported by others for *S. aureus* PDF [29].

Together, these data indicate that a pure induced-fit mechanism triggered by the binding of actinonin appears to direct the conformational change both in solution and in the crystalline state.

### Single Variants at Gly41 Exhibit Strongly Reduced Actinonin-Binding Potency and Catalytic Efficiency

When dealing with an induced-fit mechanism, knowledge of the initial O and final C state is crucial but does not provide direct information on the position of actinonin in the encounter complex or on the sequential mechanism of the transition process. We suspected that the conserved glycine-rich motif 1 (G_41_IGLAAXQ) could contribute to the flexibility required for the observed structural transition. Evidence for such flexibility comes from NMR analysis of *Ec*PDF in which a few residues show exchange cross-peaks of an additional, alternative form [38]. The most strongly affected residues are Cys90, one of the metal ligands, its neighbor Leu91, and both of the alanines within the above conserved glycine-rich motif (Figure S1B), suggesting that *Ec*PDF undergoes conformational dynamics in a similar region.

**Figure 5:**
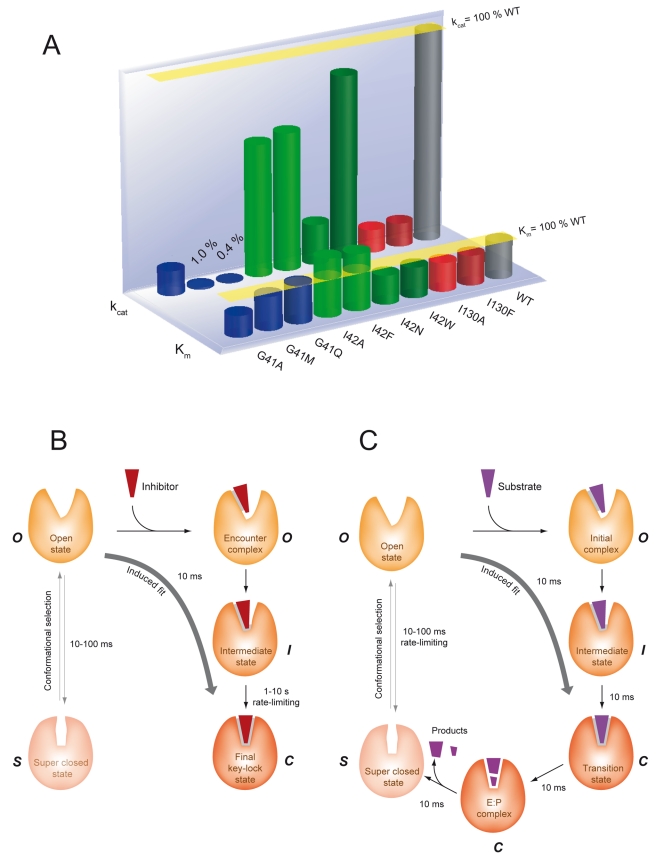
Inhibition and enzymatic reactions progress through an induced fit pathway. (A) The catalytic parameters *K_m_* and *k_cat,_* for all *At*PDF variants are provided as a percentage of the wild-type values (WT). Detailed values are presented in Table S2. (B) Schematic model for actinonin binding to *At*PDF in favor of an induced-fit pathway. PDF might exist in at least two conformational states, open (O) or closed (C). The relative abundance of each conformation would vary, depending on the enzyme type. With *At*PDF, it is likely that the most abundant form is the O one, which is the only form leading to a productive complex. The superclosed form (S) is likely to show reduced affinity for the ligand because of steric occlusion of the active site. At the initial stage, the inhibitor (shown in red) binds to *At*PDF (indicated in brown) in the O conformation. To reach the final key-lock state (productive closed conformation, C), two major and extreme pathways can be used. According to the conformational selection pathway, the inhibitor selects the C conformation. This pathway, which is represented by the dashed arrow, does not occur within the crystal. In contrast, the G41Q and G41M mutants, by providing the structure of the enzyme in intermediate conformations (I), prove the existence of the so-called encounter complex and confirm that the inhibitor binds to the enzyme when it is in the O conformation. The ligand-binding site is then reorganized to yield the C enzyme conformation, that is, the key-lock state. Indeed, the inhibitor binds to the enzyme through the induced-fit pathway. Each timescale was calculated using the data available in the text and corresponds to *t_1/2_* values deduced from the calculation of 0.693/(kinetic constant of interest). The *k_cat_* value (*k_2_*) was used to assess the timescale of catalysis in panel C, whereas, in (B), *k_4_* assesses the first step of inhibition, and *k_6_* is used in the case of the slow step. For the SO conversion (left, B), the lifetime of the minor form of *Ec*PDF was used to assess the order of magnitude (see text and [38]). (C) Schematic model for the deformylation reaction catalyzed by PDF. Since actinonin is a pseudo-peptidic inhibitor, it is likely that a peptidic substrate can bind to the PDF enzyme through an induced-fit pathway, as described in (B). The key-lock state represents a transition state in which the N-formylated substrate is deformylated to yield the final reaction product.

To unravel the dynamics of the recognition process, we surmised that it should be possible to freeze the conformational change along the pathway by introducing selected, minor variations within the above-mentioned crucial residues involved in the collective motion. In this respect, site-directed mutagenesis of *At*PDF was performed on Gly41, Ile42, and Ile130. Single substitutions were made at Gly41 (G41A/Q/M), Ile42 (I42A/F/N/W), and Ile130 (I130A/F), and the variants were purified and characterized. These mutant proteins showed no change in overall stability, as evidenced by DSC experiments (unpublished data). However, two variants of G41, G41Q and G41M, showed dramatic effects; the *k*_cat_/*K*_m_ values were reduced by three orders of magnitude due to large decreases in the *k*_cat_ values compared to the WT enzyme (Figure 5A and Table S1). The reduced *k*_cat_/*K*_m_ values suggest an altered ability of these variants to attain the final enzyme-transition state complex and, as a result, to give rise to possible states different from the final E:I* complex. Substitutions at positions 42 and 130 only caused small reductions in the *k*_cat_ values (Figure 5A, Figure S2C, and Table S1). The actinonin-binding potency of both G41 variants was also greatly reduced (Table S1 and Figure S2B). The time-dependent inhibition by actinonin of the most active variants was then studied (Table S3). The half-lives of the final complexes—as assessed by comparison of the 1_/_ *k_6_* values—were always significantly smaller (Table S3), suggesting that the conformational change induced by actinonin binding still occurred, but the C state is destabilized relative to the O state in the mutants compared to the WT. Accordingly, actinonin strongly stabilized almost all of the variants; *T*_m_ was increased by more than 20°C. This differs from the G41M and G41Q variants, which both showed increases in the *T*_m_ of only 12°C, consistent with reduced binding potency (Table S1).

### Conformational Changes of Gly41 Variants Are Affected On-Pathway

The two most interesting variants, G41Q and G41M, could be crystallized under the same conditions as the WT protein. In the case of G41Q, the structure of the apo-protein did not show any modifications compared to the WT structure and remained in an O conformation (Figure 2B; “O” zone). In contrast, the 3D structure of the G41M variant showed that the asymmetric unit was composed of two molecules with distinct structures. One molecule (chain A) is in the O state and is similar to the structures of the WT and the G41Q variant (Figure 2B; zone “O”). The second molecule (chain B) is in a C state, closer to that observed for the WT chain in the presence of actinonin (“C”), a so-called “superclosed” state (Figure 2B; zone “S”), suggesting that the substitution modified the equilibrium between the two states in solution either (i) at the step of protein synthesis by providing two conformers, the inter-conversions of which are blocked due to steric hindrance brought by the new bulkier side-chain at position 41, or (ii) by dramatically unbalancing the free inter-conversion between the O and S conformers towards the S state. Ringer analysis indicates that in the free G41M variant, many residues show evidence for unmodeled alternate conformers—including positions 58, 42, and 130—in keeping with the second hypothesis.

For all variants of position G41, addition of actinonin to the crystal (Figure 3 and Figure S6) induced a closure of the protein within the crystal. Nevertheless, as expected from in silico graphic modeling followed by energy minimization, the occurrence of a bulky side chain at position 41 prevented the completion of the closure in the presence of the ligand and, hence, the formation of the hydrogen bond between the backbone nitrogen of Ile42 and actinonin. This finding is consistent with the strongly reduced *T*_m_ of the complex of the variants with actinonin compared to WT as measured by DSC. Remarkably, both S and O forms of the G41M apo-structures in the asymmetric unit of the crystal yielded a unique intermediary structure (“I” state) upon actinonin binding (r.m.s.d. between the molecules is <0.25 Å; see also Figure 2B, zone “I”). In this case, it is likely that the induced-fit mechanism drives the equilibrium by capturing only the O population and closing it to an intermediary step, thus depleting the pool of O conformers that is shifted sequentially back from the remaining pool of S conformers and allows the complete binding of actinonin to the enzyme.

In line with the rational design of the PDF mutants, the extent of the structural differences suggests that the underlying motions are dependent on the length of the side chain (Figure S8). Together, these data account for the reduced catalytic rate, as the hydrogen bond is strictly required for the substrate to be efficiently cleaved by PDFs (Figure S8A) [54]. Therefore, from both structural and kinetic analyses, each substitution most likely reproduces intermediates along the pathway that lead to the closure of PDF around its substrate (Figure S2B).

### Conformational Changes of Gly41 Variants Recapitulate Closing Intermediates

Analysis of the structures allows us to propose the following sequence of atomic events (Figures 3 and 2B and Figure S6). To name the various sites of the ligand and subsites of PDF, we will use the usual nomenclature found in [55], which defines the various binding pockets of a protease, where P1' is the first side chain at the C-terminal side of the cleavage site and its binding pocket is S1', also referred to as the hydrophobic pocket in the case of PDF. First, actinonin aligns along the S1' pocket to form the encounter complex, which shifts the Ile130 side chain to avoid steric hindrance in the S1' pocket, promotes rotation of the Ile42 side chain, and finally rearranges the phenyl group of Phe58. These events achieve an optimal hydrophobic S1' pocket conformation (Figure 3), and the concomitant closure leads to the formation of a hydrogen bond between the first carbonyl group of actinonin and the backbone nitrogen of Ile42. The initial N-O distance is reduced from 5 Å to 2.8 Å, which is an optimal value for hydrogen bonding (Movie S2 and Figure S8B). Thus, the primary driving force for the active site closure appears to be the P1':S1' hydrophobic interaction. The C state is ultimately locked by the super-β-sheet hydrogen bonds extending across the ligand, including those involving Ile42. The ΔΔG_binding_ value (2.2–2.4 kcal/mol, Figure S8B), as calculated from the *K*_d_ values for actinonin binding to wild-type (WT) and G41M and G41Q, is consistent with the loss of a hydrogen bond that also contributes to the conformational stability of the protein [56],[57]. Thus, this bond contributes to the major binding free energy difference between the two complexes (3.1 kcal/mol; Figure S8B, Tables S1 and S3, and [29]). Interestingly, the above ΔΔG_binding_ values also correlate with the ΔΔG_ES_ values derived from the *k_cat_*/*K_m_* and *k_cat_* measurements [19]. This dataset strongly correlates with the gyration and van der Waals radii of the side chain at position 41 as well as the N-O distance between the first carbonyl group of actinonin and the backbone nitrogen of Ile42 (Figure S8). These results suggest that the capacity of both G41M and G41Q variants to form the transition state is a consequence of their inability to reach the fully closed state.

Thus, our study of the designed Gly41 mutant enzymes reveals that, in addition to the initial and final states observed for the WT enzyme, the conformations of the Gly41 variants correspond indeed to on-pathway intermediates, thus providing snapshots along the trajectory from the O to the C state of the enzyme (Figures 2B and 3). The 3D structure of the variants in the absence of ligand is similar to that of WT, and a strict correlation exists between the completeness of the conformational change and both binding potency and catalytic efficiency. This suggests that both events require complete protein closure to generate a productive complex. The strong stabilization of *At*PDF by actinonin (Figure 1D) closely mimics what occurs with its natural substrates when it reaches the transition state [34],[58]. Indeed, as expected, the enzyme facilitates the final C conformation by lowering its final energy [6]. Optimal arrangement of the S1' pocket (Figure 3) proceeds along the reaction process towards the final C conformation, triggering the alignment of reactive groups in an optimal arrangement for ligand recognition. Upon binding, actinonin alters the thermodynamic landscape for the structural transition between the O and C states. This ligand is a potent inhibitor because it can trigger the above sequence of events similar to the substrate, but unlike the substrate, it is non-hydrolyzable. Thus, by mimicking the transition state and being non-hydrolyzable (Figure 1B), the final C complex is long lasting.

### Ligand-Induced Conformational Closure Is Initially Triggered by the Binding of the P1' Group in the S1' Pocket

**Figure 6:**
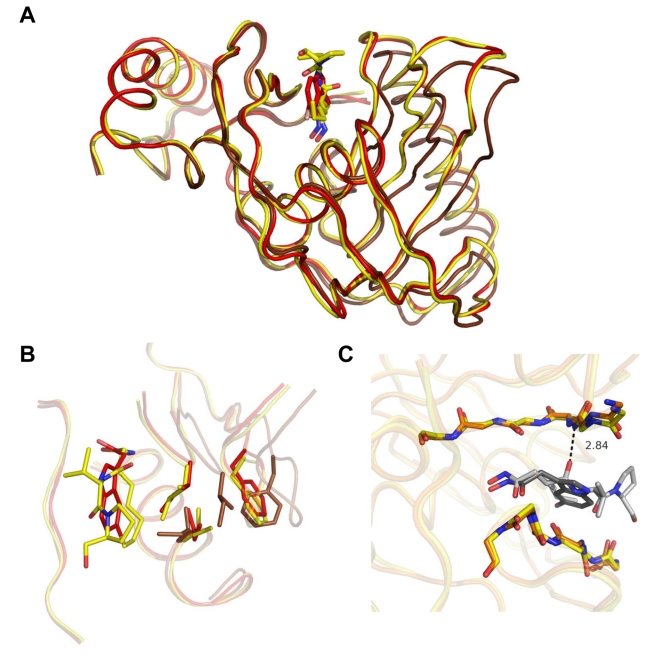
Effect of *6b* binding on the conformation of key residues of PDF. Superimposition of free, ***6b***-, and actinonin-bound *At*PDF indicated in brown, red, and yellow, respectively. (A) Molecule A in the three models was superimposed, resulting in an r.m.s.d. of 0.9 Å for 100% of the C*α*. Actinonin is shown in yellow and ***6b*** in red. (B) Conformation of key residues Ile42, Phe58, and Ile130 in the different complexes and in unbound WT *At*PDF. Actinonin is shown in yellow and ***6b*** in red. (C) A detailed view of the *At*PDF ligand-binding site for both actinonin and ***6b*** complexes, which are indicated by sticks and are superimposed. The two ligands are colored in pale and dark grey, respectively. The hydrogen bond made by actinonin only is shown.

Given the similarity between actinonin and natural substrate binding, the very slow kinetics of inhibitor binding (10-s time-scale) remains puzzling compared to the 10 ms required for catalysis (deduced from the *k_cat_*). This finding could be explained as a conformational effect during the formation of the hydrogen bond, aligning the substrate as an additional beta-sheet and eventually stabilizing the entire enzyme-ligand complex. The significantly longer time needed to reach the most stable state compared to the substrate would most likely be due to the presence of the flexible and one carbon longer metal-binding group in actinonin (i.e., hydroxamate versus formyl, Figure 1B). This suggestion is in line with the overall data obtained when we investigated more deeply the role of the first carbonyl group of the ligand. This group is well known to exert a crucial effect in both productive and unproductive ligand binding (i.e., substrate and inhibitor) [54]. In this respect, we studied the binding of compound ***6b*** (Figure S5B), a PDF ligand that does not exhibit a reactive group at this position [49]. We observed that this compound binds strongly to both *Ec*PDF (K_I*_  = 63±6 nM) and *At*PDF (K_I*_  = 400±35 nM) but, unlike actinonin, does not display slow, tight binding as K_I*_  =  *K_I_*. This impact on binding is consistent with the absence of the hydrogen bond involving the first carbonyl group of the ligand. The 3D structure of *At*PDF was determined after soaking the compound in crystals of the free, open *At*PDF form. Upon binding, ***6b*** induced a complete conformational change, identical to that observed with actinonin (Figures 2B and 6A; “O” state). This result further suggests that the conformational change is not induced initially by the formation of this hydrogen bond and that the encounter complex is primarily driven by the fit within the S1' pocket. This also reveals that the timescale of the large conformational change is several orders of magnitude faster than the kinetics of slow binding and fully compatible with both the first step of actinonin binding (*k_4_*  = 140 s^−1^; see Table 1) and the catalytic rate of the substrate (*k_ca_*_t_  = 37 s^−1^; see Table 1 and Table S3). The 3D structure also revealed that both the P1' and the hydroxamate groups are bound similarly to the corresponding groups of actinonin (Figure 6B). As expected, no additional bonding occurs, especially around the backbone nitrogen of Ile42 (Figure 6C).

Taken together, these data allow us to conclude that the conformational change observed upon ligand binding is triggered primarily by binding in the S1' pocket. As revealed by the binding of ***6b***, the one carbon longer metal-binding group fits, immediately upon recognition of the P1' group, in the S1' pocket and forms a bidentate complex with the metal cation, mimicking the transition state as a result. Thus, the active site is very confined and rigid due to the presence and length of the hydroxamate group (compare right and left panels in Figure 1B). As a result, compared to the complex made with the substrate, it is likely that the formation of the hydrogen bond involving the carbonyl of actinonin and the backbone nitrogen of Ile42 becomes strongly rate-limiting (*k_5_*  = 0.044 s^−1^; Table 1). Once this hydrogen link is locked, the uncleavable bond, mimicking the labile formyl group at the transition state, stabilizes the enzyme-inhibitor complex, making it long-lasting (*k_6_*  = 0.0006 s^−1^; Table 1) and providing a mechanistic explanation for the slow-binding effect that involves both large and fine conformational changes. The large conformational change is similar to the one occurring with the substrate, whereas the second is more subtle and locks the hydrogen bond involving the backbone nitrogen of Ile42. The second step is rate-limiting with some transition state analogs such as actinonin (Figure 5B and C).

### Proper Positioning of the Carbonyl Group Is Required to Stabilize the Complex at S1'

Compound ***21*** corresponds to another interesting derivative designed to probe the impact of the peptide bond in PDF binding [49]. In addition to the hydroxamate group, this compound features both a hydrophobic benzyl group at P1' and a reverse peptide bond. Compound ***21*** shows modest but significant inhibitory potency to *At*PDF1B (K_I_*  = 400±37 nM), confirming the crucial role of the peptide bond in PDF binding. After soaking with crystals of apo-*At*PDF, compound ***21*** could be detected in high-resolution electron density maps (Figure S9A). Unlike ***6b***, ***21*** did not bind the active site of the enzyme but an alternative pocket at the surface of the protein (Figure S9B). A docking study performed with *Ec*PDF had previously revealed this alternative binding pocket (Figure S9C; [59]).

The aforementioned data indicate that the occurrence of a S1'-binding group placed in the unfavorable context of a reverse peptide bond does not stably promote binding at the active site of *At*PDF. Upon binding of ***21***, the 3D structure of both molecules of the asymmetric unit remain in an O conformation (r.m.s.d. <0.2 Å with respect to the apo-structures in the “O” state). This finding suggests that only the binding of compounds entering the S1' pocket, such as actinonin or ***6b***, induces conformational change, in keeping with the crucial role of the P1' group if located in the frame of a classic peptide bond. Moreover, we noticed that the binding pocket of ***21*** was located on the rear side of the true S1' pocket and induced a weak modification of the P1' hosting platform (Figure S9D). Indeed, when crystals of the ***21***:*At*PDF complex were soaked in actinonin, the final 3D structure no longer showed evidence of compound ***21*** occupancy greater than 5%. Instead, this structure revealed both actinonin and closing of the protein (Table S2). The r.m.s.d. between this structure and that obtained directly with actinonin was less than 0.2 Å; the actinonin position was virtually identical, indicating that the protein had retained full capacity for binding actinonin and closing despite the presence of compound ***21***. We conclude that actinonin does compete with ***21*** because of the overlap at P1' of *At*PDF1B (Figure S9C). As the actinonin S1' subsite strongly mimics that of a true substrate, this result also explains the inhibitory behavior of ***21*** towards *At*PDF.

## Discussion

Although PDF catalysis has been extensively studied and the mechanism has been elucidated [34], how the enzyme achieves the catalytically competent state remains unknown. Here, we provide insight on how the enzyme might reach a catalytically competent conformation, demonstrating that the reactive groups move into proximity to promote catalysis (Figures 2B and 5C). We suggest that the motions of the catalytic centre starting with free ligand-PDF favor a final configuration that is optimal for binding and/or catalysis (Figures 1B, 2B, and 5B and C). In our model, we propose that free PDF might exist in at least two conformational states, that is, open (O) or super-closed (S). The relative abundance of each conformation varies by enzyme type and incubation conditions, explaining why both conformations have not been trapped thus far. In the case of *At*PDF, it is likely that the most abundant form corresponds to an O state, which is the form that leads to a productive complex. Indeed, in the NMR spectra for *Ec*PDF, a few residues show exchange cross-peaks from an additional, alternative form [38]. The most strongly affected residues are Cys90, one of the metal ligands, its neighbor Leu91, as well as Ala47 and Ala48 on the facing strand. This suggests that *Ec*PDF exists in at least two conformations (“S” and “C”; see Figure 2B), which undergo slow interconversion on the NMR timescale. The 3D structure of the major conformation (75%, lifetime 300 ms) could be solved at high resolution, but the structure of the minor form (25%, lifetime 100 ms), which exhibits very weak signals, could not be solved [38]. This conformation appears to correspond to that of the complex obtained with the product of the reaction (Met-Ala-Ser). A very similar situation—although more balanced between the two states—appears to occur in the case of variant G41M, suggesting that a mechanism involving conformational selection followed by induced fit is a general model for PDF and that *At*PDF is a specific case where population shift virtually does not occur as the free enzyme is completely in the O conformation. This is also in line with data obtained with *L. interrogans* PDF (*Li*PDF), which reveal conformers in both the S and C states (see Figure 2B) and suggest a population-shift mechanism [43]. It is interesting to note that *Li*PDF is a poorly active PDF [60]. According to the representation shown in Figure 2B, *Plasmodium falciparum* PDF (*Pf*PDF), a poorly active PDF [61], was retrieved only in the S state. Finally, weak decompaction of the structure of *Bacillus cereus* and *Staphylococcus aureus* PDFs in the presence of actinonin have been described [45],[46]. These examples suggest that the enzyme is trapped in the S conformer in the free state and converts to the C conformer when bound to actinonin, suggesting that the S conformer is overrepresented in solution compared to the O state, unlike *At*PDF.

This study of *At*PDF—including 10 different crystal structures of apo- and complexed enzyme variants—reveals the 3D structure of a PDF in at least four distinct states. This includes the O form, the occurrence of which is crucial for catalysis, as it is the active form. Here, we propose that the transition from the O to the C state is directly induced by the ligand. Indeed, the O form, which is captured in the crystal, undergoes closure directly upon ligand binding in our soaking experiments. Progression to this closure involves intermediary states (“I”) similar to those observed with variants G41Q and G41M in the presence of actinonin (see Figure 2B). Extrapolating the situation to catalysis, which occurs in the crystalline states of PDF, it is likely that hydrolysis of the substrate frees the enzyme in its S state, which in turn needs to open to accommodate a new substrate (Figures 2B and 5C). This is well illustrated in the 3D structure of *Ec*PDF complexed with a product of the reaction, obtained after co-crystallization of the enzyme with the substrate in a closed conformation [34]. The S free form is likely to exhibit a slower on-rate for the ligand (*k_3_*) compared to the O form because of steric occlusion of the active site (Figure S10). In support of this hypothesis, recent data show that the 3D structure of a C-terminally truncated, poorly active version of *At*PDF is in the C conformation in the unbound state, although crystallized under conditions identical to ours [62],[63]. This structure is similar to that of chain B, one of the two molecules of the asymmetric subunit of variant G41M (Figure 2B). This suggests that alterations remote from the active site significantly unbalance the equilibrium between the two conformers, thus altering the efficiency of the reaction (Figure 5C). As the S version corresponds to a significantly less active version of *At*PDF compared to that reported in our present work, this further confirms that, compared to the O state, the S state has a significantly weaker propensity to bind substrate or a close mimic ligand, such as actinonin. Comparison of the 3D structures of the free-closed and the ligand-bound-closed forms reveals some differences responsible for the slight steric reduction of the active site of free-closed *At*PDF1B with respect to that of the actinonin-*At*PDF1B complex (Figure S10A), including the side chain of Ile42 burying the S1' binding pocket (Figure S10B). Overall, these data suggest that an S form might exist under the free state but that it would feature a *k_3_* value with respect to the ligand that is significantly weaker than that of the O form, which would strongly slow down the reaction or the binding as a result.

With the interaction scheme proposed in our model (Figure 5B and C), the ligand/substrate binds more easily to the O form and induces the optimal conformation of the enzyme to reach the transition state, thus allowing the reaction to be efficiently catalyzed. In the final model (Figure 5C), there is both conformational selection and induced fit subsequently involved in line with the recently proposed existence of such mixed mechanisms for other enzymes [15],[16]. Nevertheless, in our model (Figure 5C), we suggest that induced fit is the primary mechanism, as it provides energy input from the ligand, which eventually drives the enzyme towards the productive key-lock complex. Unambiguous distinction between the relative contributions of the two mechanisms is deduced from the observation that *k_ob_*_s_ is a saturable function of actinonin with various PDF, including *Ec*PDF, *Bs*PDF, *At*PDF (Figure 4B and C), and *S. aureus* PDF [12],[16],[29],[49].

Using crystallographic reconstruction analysis involving enzyme variants, motions of small mobile loops and movie reconstructions of snapshots of catalytic events have been previously documented [1]–[3],[64]–[66], often by visualizing the binding of unnatural inhibitors and not necessarily mimicking closely the substrate and transition state as actinonin does [67],[68]. However, only a few examples make use of soaking conditions of a crystal to promote the motion and show the importance of induced fit [1],[69]. None of these data show a motion of the amplitude revealed here with PDF and a large stabilization of the complex involving the formation of the four-stranded β-sheet superstructure and the entire N-domain of the enzyme. Compared to previous crystallographic analyses, our work integrates biophysical, computational, and kinetic analyses to reconstruct the whole picture, allowing a better understanding of the slow-binding mechanism.

While our work primarily focused on an induced-fit mechanism of enzyme inhibition and catalysis, it should be emphasized that this phenomenon is also applicable to the broader area of receptor-ligand interactions. For example, in all cases where conformational change mechanisms have been proposed for kinase inhibitors without supporting experimental data [12],[26], further experimental work must be provided to clarify the precise mechanism. We expect this will have important implications on how one conducts future drug-discovery efforts against such enzymes [70].

## Materials and Methods

### Protein Expression and Purification

Expression and purification of mature *Arabidopsis thaliana* PDF1B and all variants (i.e., *At*PDF) were derived from the previously described protocol [37]: the lysis supernatant after sonication was applied on a Q-Sepharose column (GE Healthcare; buffers A and B as described containing 5 mM NiCl_2_) followed by Superdex-75 chromatography (GE Healthcare) using buffer C consisting of buffer A supplemented with 0.1 M NaCl. For crystallization experiments, the protein was purified further. The sample was concentrated on an Amicon Ultra-15 centrifugal filter unit (Millipore Corp.) with a 5-kDa cut-off and applied to a MonoQ HR5/5 column (GE Healthcare) previously equilibrated in buffer A (50 mM Hepes, pH 7.5, and 5 mM NiCl_2_). Elution was performed with a 50-mL gradient from 0% to 100% buffer B. The buffer of the pooled purified *At*PDF1B was exchanged using a PD-10 desalting column (GE Healthcare) to yield a protein solution in 50 mM Hepes, pH 7.5, 0.1 M NaCl, and 5 mM NiCl_2_ (buffer C). The protein was concentrated on an Amicon Ultra-15 centrifugal filter unit. The resulting *At*PDF1B preparation was frozen in aliquots and stored at −80°C (for crystallization purposes) or diluted 2-fold in 100% glycerol and stored at −20°C (for enzymatic purposes). The typical yield was 5–10 mg *At*PDF per liter of culture. All purification procedures were performed at 4°C. Samples of the collected fractions were analyzed by SDS-PAGE on 12% acrylamide gels, and protein concentrations were estimated from the calculated extinction coefficients for each variant.

Site-directed mutagenesis of *At*PDF sequence in plasmid pQdef1bΔN [36] was carried out using the QuickChange Site-Directed Mutagenesis Kit (Stratagene).

### Enzymology

Assay of PDF activity was coupled to formate dehydrogenase, where the absorbance of NADH at 340 nm was measured at 37°C as previously described [71]. For measurements of classical kinetic parameters (i.e., *K*_m_ and *k*_cat_), the reaction was initiated by addition of the substrate *Fo*-Met-Ala-Ser to the mixture containing purified enzyme in the presence of 1 mM NiCl_2_. The kinetics parameters were derived from iterative non-linear least square calculations using the Michaelis-Menten equation based on the experimental data (Sigma-Plot; Kinetics module). For determination of kinetic parameters related to actinonin, the reaction mixture contained 750 µM NiCl_2_. In some cases, the mixture containing PDF and actinonin was incubated for 15 min at 37°C before kinetic analysis, which was initiated by the addition of substrate. The same protocol was used to determine the dissociation constant of actinonin [*K*_I_*  =  *k_4_*/(*k_3_*+*k_3_k_5_/k_6_*)], but the initial reaction velocities were measured with varying concentrations of *Fo*-Met-Ala-Ser and actinonin. The data were then calculated according to the method of Henderson, which can be used to determine the dissociation constant of the tight-binding competitive enzyme inhibitor [28],[49],[72] by varying both the inhibitor and substrate concentrations. To determine *K*_I_, *k*_5_, and *k*_6_, the reaction was initiated by the addition of enzyme as previously described [29],[49]. *K*_I_*_app_ measurements were used for comparative studies of *At*PDF variants (Table S3) at a concentration of 2 mM substrate by varying the concentration of actinonin. *K*_I_*_app_ is the slope of the *v_[Actinonin]_*/*v_0_* line curve. *k_obs_* was fitted from the kinetic data without preincubation with *v_I_*  =  *v_s_* + (*v_0_* − *v_s_*)e^−*kobs*t^ where *v_I_* is the observed velocity at a given concentration of inhibitor I, *v_0_* is the velocity, and *v_s_* is the steady-state velocity [18]. From the set of values obtained at various concentrations of I, *k_5_* and *k_6_* could be derived using *k_obs_*  =  *k_6_* + *k_5_*[I]/(K_I_ + [I]). By choosing a set of values with *k_obs_*>>*k_6_*, 1/*k_obs_*  = 1/*k_5_*(K_I_/[I] +1) and 1/*k_obs_*  =  f(1/[I]) is expected to be a straight line in case of induced fit whose positive slope corresponds to 1/*k_5_*. *k_6_* was derived from equation *k_6_*  =  *k_5_*/(*K_I_*/K_I_*−*1*) [18],[19].

### Microcalorimetry

ITC experiments were performed using a VP-ITC isothermal titration calorimeter (Microcal Corp.). Experiments were performed at 37°C. For each experiment, injections of 10 µL actinonin (180 µM) were added using a computer-controlled 300 µL microsyringe at intervals of 240 s into the Ni-*At*PDF variant solution (5 to 10 µM, cell volume  = 2.1 mL) dissolved in buffer C with stirring at 310 rpm. A theoretical titration curve was fitted to the experimental data using the ORIGIN software (Microcal). This software uses the relationship between the heat generated after each injection and Δ*H*° (enthalpy change in kcal/mol), *K*_A_ (the association binding constant in M^−1^), *n* (number of binding sites per monomer), total protein concentration, and free and total ligand concentrations. The thermal stability of the WT and variants of Ni-*At*PDF1B was studied by DSC using VP-DSC calorimetry (Microcal Corp.). DSC measurements were made with 10 µM protein solutions in buffer C. The actinonin concentration was 20 µM. The same buffer was used as a reference. All solutions were degassed just before loading into the calorimeter. Scanning was performed at 1°C/min. The temperature dependence of the partial molar capacity (Cp) was expressed in kcal/K after subtracting the buffer signal using Origin(R) software.

### Crystallization and Soaking Experiments

Crystallization conditions were screened by a robot using the sitting drop vapor diffusion method. Crystals were obtained and optimized at 20°C with 15%–20% PEG-3350 and either 0.1 or 0.2 M zinc acetate. The drops were formed by mixing 2 µL of a solution containing 2 to 4 mg/mL protein and 2 µL of the crystallization solution. Crystals were soaked for 24 h by adding actinonin to the crystallization drops at a final concentration of 5 mM. Cryoprotection was achieved by placing crystals for 30 s in a solution that was composed of 20% PEG-3350 and 0.2 M zinc acetate, supplemented with 5%, 10%, and 15% glycerol. Crystals were then directly flash frozen in liquid nitrogen using cryoloops (Hampton Research). Crystals were also grown under conditions described for the C-terminally deleted, weakly active version of *At*PDF [63].

### X-Ray Diffraction Data Collection

Data collections were performed at 100 K at the European Synchrotron Radiation Facility (Grenoble, France) on station ID29, FIP-BM30A, ID14-1, and ID23-2, and at SOLEIL (Gif-sur-Yvette, France) on station PROXIMA1. In each case, a single crystal was used to collect a complete dataset. Data were processed and scaled using XDS software [73]. Two crystal forms were encountered with different cell parameters. In each case, *b* parameter was nearly equal to *a*, and data could be indexed into two space groups, *P2_1_2_1_2_1_* or *P4_3_2_1_2*. The data are shown in Table S2.

### Structure Determination and Refinement

The structure of free *At*PDF was solved by molecular replacement with Phaser [74] followed by a rigid-body refinement by CNS [75] using coordinates from the *Plasmodium falciparum* PDF (PDB code 1RL4) [76] as a search model. The structures of actinonin-bound proteins—that is, WT and mutants—were solved using rigid-body refinement by CNS of the free *At*PDF structure. The ten final models were obtained by manual rebuilding using TURBO-FRODO [77] and combined with refinement of only calculated phases using CNS and Refmac [78] software. No non-crystallographic symmetries were used. Quality control of the three models was performed using the PROCHECK program [79]. To probe for alternative conformers, Ringer was used [53]. Ringer is a program to detect molecular motions by automatic X-ray electron density sampling, and can be accessed at http://ucxray.berkeley.edu/ringer.htm.

### Accession Numbers

PDB codes for the PDF structures presented within this manuscript are as follows: 3M6O, 3PN2, 3M6P, 3O3J, 3PN3, 3PN4, 3PN5, 3M6Q, 3PN6, and 3M6R. UniProtKB accession numbers for other PDF studied are P0A6K3 (EcPDF) and O31410 (BsPDF).

## Supporting Information

Figure S1 Alignment of PDF sequences and secondary structures. (A) PDF1B from *Arabidopsis thaliana* (*At*PDF1B) is compared with bacterial type 1B (*Ec*PDF and *Li*PDF), pathogenic protozoa (*Pf*PDF1B), eukaryotic mitochondrial PDF1A from *A. thaliana* (*At*PDF1A), and bacterial type 2 (*Bs*PDF2). This figure was created with ENDscript [80]. The sequence alignment was realized with the algorithm muscle included in ENDscript, and modified according to the superimposition of structures. The blue frames indicate conserved residues, white characters in red boxes indicate strict identity, and red characters in yellow boxes indicate homology. The secondary structures at the top (α-helices, 3_10_ helices, *β*-strands, and *β*-turns are shown by medium squiggles, small squiggles, arrows, and TT letters, respectively) were predicted by DSSP [81]. Relative accessibility (acc) of subunit A is shown by a blue-colored bar below sequence. White is buried, cyan is intermediate, and blue with red borders is highly exposed. A red box means that relative accessibility is not calculated for the residue, because it is truncated. Hydropathy (hyd) is calculated from the sequence according to [82]. It is shown by a second bar below accessibility: pink is hydrophobic, grey is intermediate, and cyan is hydrophilic. Motifs 1 (_41_G*φ*G*φ*AA*X*Q_48_), 2 (_89_EGCLS_93_), and 3 (_133_HE*φ*DH_137_), where *φ* is a hydrophobic amino acid, are labeled by red stars below the sequence alignment. To simplify the nomenclature, *At*PDF1B is referred to as *At*PDF throughout the text. (B) Topology cartoon of *At*PDF, free (left) or actinonin bound (right), in the same color code as (A). Actinonin (represented by the yellow arrow) binding to the ligand binding site allows the linkage of the two distinct *β*-sheets into one single *β*-sheet, by mimicking an additional *β*-strand. PDB sum (http://www.ebi.ac.uk/thornton-srv/databases/pdbsum/) was used. (C) 3-D structure of *At*PDF is represented showing the position of the residues discussed in the text, indicated in red.
(EPS)

Figure S2 Microcalorimetric titration of *At*PDF with actinonin. Data were obtained at 37°C by an automated sequence of 28 injections of 180 µM actinonin from a 300 µl syringe into the reaction cell, which contain 9.85 µM *At*PDF. The volume of each reaction was 10 µl, and injections were made at 240 s intervals. Top, raw data from the titration. Each peak corresponds to the injection. Bottom, the peaks in the upper panel were integrated with ORIGIN software and the values were plotted versus injection number. Each point corresponds to the heat in µcal generated by the reaction upon each injection. The solid line is the curve fit to the data by the Origin program. This fit yields values for *K*_d_. Experiments were done with wild type protein and others variants, and gave similar raw data and curve fit. (A) WT; (B) variant G41M; (C) variant I42W.
(EPS)

Figure S3 Binding of actinonin to *At*PDF does barely modify the crystal packing. (A) Crystal pack of the two complexes: open, free complex (left) and bound to actinonin (right) (B). Non-crystallographic contacts into asymmetric unit are not modified by closing movement of the protein due to actinonin binding, except for zinc atom number 6. This metal ion is coordinated by side chains of Asp40 and Glu63, and water molecules, Asp40 and Glu63 being hydrogen bonded by side chain of Lys38 of the other subunit of the asymmetric unit. With the closing movement of the protein into the crystal, Cα of Asp40 shifted by 3.1 Å and its side chain flipped by 90°. Therefore, it does no longer participate to the coordination shell of this Zn^2+^ ion. However, it is still hydrogen bonded by Lys38 from chain B.
(EPS)

Figure S4 Binding of actinonin to *At*PDF closely mimics both actinonin and product binding to *Ec*PDF. Superimposition of *Ec*PDF and *At*PDF bound to either actinonin (1LRU PDB code, panel A) or Met-Ala-Ser (1BS6 PDB code, panel B), the product of the reaction. The r.m.s.d. value is 1.11 Å for 151 Cα superimposed.
(EPS)

Figure S5 The ligand binding site of *At*PDF. This picture shows the residues of *At*PDF that are in contact with actinonin (left) and ***6b*** (right) according to the 3-D structure; this should be compared to the similar scheme shown in Figure 1B for *Ec*PDF.
(EPS)

Figure S6 Electronic densities of the moving side-chains and of actinonin at the binding site in some variants of *At*PDF. Actinonin and selected residues (G/Q/M41, I42, F58, and I130) are drawn in stick and are shown in their F*_O_*–F*_C_* electron density omit maps contoured at 2σ, in free wild-type *At*PDF (two crystallization conditions, WT1 and WT2), and ligand-bound WT (actinonin, ***6b*** and ***21***), G41Q, and G41M variants.
(EPS)

Figure S7 Only few residues show alternative conformation in *At*PDF. Alternative conformers in the crystalline state of *At*PDF. Ringer plots of electron density (r) versus χ^1^ angle for representative residues of the 3-D apostructure of *At*PDF. Data were obtained with the 3M6O dataset (see Table S1). The secondary peaks in the Ile residues are observed because Ile is a branched amino acid. To evidence an alternative conformation with Ile, three peaks should be observed.
(EPS)

Figure S8 Impact of induced fit on the binding free energy of actinonin depends on the capacity to stabilize a hydrogen bond with PDF. (A) The gyration radii [83] of the side chain occurring at position 41 is displayed with black squares and compared to the *k_cat_/K_m_* values (grey bars). (B) The distance between the NH of I42 and the CO of actinonin was measured in each case. The percentage of the distance required to make a hydrogen bond (2.8 Å) is reported (dark squares). The difference of binding free energy (ΔΔG_binding_) between the open, free state and the variants closed complexes of the G41 variants are displayed as grey bars. The values were calculated as follows. For the WT, it corresponds to the *RT* ln(K_I*_/K_I_) value [29], where *R* is the ideal gas constant and *T* is the temperature in Kelvin. *RT* is 0.616 kcal.mol^−1^ at 37°C. For the G41M and G41Q variants, the ΔΔG_binding_ corresponds to *RT* ln(*K_I-G41variant_*/*K_D-WT_*). The obtained values are similar to that obtained if the *k_cat_/K_m_* substitutes the *K_D_* value in the calculation (ΔΔG_binding_ =  *RT* ln(*k_cat_/K_m –G41variant_*/*k_cat_/K_m –WT_*).
(EPS)

Figure S9 Compound ***21*** does not bind *At*PDF1B at S1'. (A) ***21*** is shown in ball-and-stick format in its F*_O_*–F*_C_* electron density omit map contoured at 2*σ*. (B) Binding site of ***21*** into *At*PDF1B is detailed. Red and blue residues indicate residues that accommodate the “phenylalanine” and “trimethyl” groups of ***21***, respectively. (C) Overall view of ***21*** binding site (left). Molecular surface of *At*PDF is represented, as well as ***21*** in ball-and-stick format. Residues belonging to the ***21*** binding pocket are colored in orange. For comparison, molecular surface of *Ec*PDF (PDB code 1G2A) in the same orientation is also represented, with residues forming the new ligand binding pocket colored in orange. Actinonin is represented in ball-and-stick format and is seen through the molecular surface of each PDF. (D) Ball-and-stick representation of the interaction network around compound ***21***. The metal cation is shown as a grey sphere.
(EPS)

Figure S10 Poorly active versions of *At*PDF are in a closed conformation incompatible with actinonin binding. (A) Free and close *At*PDF were superimposed as in Figure 1C and are figured in brown and yellow, respectively. Both the G41M (chain B, shown in orange) and the free C-deleted weakly active *At*PDF versions ([63], colored in purple, PDB entry code 3CPM) were superimposed, to the two structures, showing that they both fit better to the ligand-bound full-length close form than to the free open form, but that the closure is further pronounced, burying the entrance to a ligand. (B) Close-up showing that the shape of the S1' pocket of the poorly active closed versions make it poorly available to P1' recognition (see circled Ile142 and Ile130 side chains).
(EPS)

Table S1 Catalytic properties of *At*PDF. Nm, not measurable; ND, not determined; WT, is wild-type. ^a^Kinetic constants were determined using the coupled assay as indicated in Materials and Methods with substrate *Fo*-Met-Ala-Ser, in the presence of 100 nM enzyme variant and 750 µM NiCl_2_, at 37°C. The relative value of *k*_cat_/*K*_m_ for wild-type *At*PDF was set at 100%. ^b^Data correspond to the binding constant of actinonin as obtained either from ITC or from enzymatic analysis when indicated with an asterisk. ^c^Data from Table S3. ^d^Gyration radii are from [83].
(DOC)

Table S2 Crystallographic data and refinement statistics. Values in parentheses are for the outer resolution shell. ^a^R_sym_ (I)  =  Σ*_hkl_Σ_i_*|*I_hkl,i_* − <*I_hkl_*>|/Σ*_hkl_Σi*|*I_hkl,i_*|, where <*I_hkl_*> is the mean intensity of the multiple *I_hkl,i_* observations for symmetry-related reflections. ^b^R_work_  = 100× (Σ*_hkl_*|Fobs − Fcalc|/Σ_hkl_|Fobs|). R_free_ is a test set including ∼5% of the data. ^c^Percentage of residues in most-favored/additionally allowed/generously allowed/disallowed regions of the Ramachandran plot. ^d^Compound ***21*** was added first, and actinonin afterwards.
(DOC)

Table S3 Kinetic parameters for inhibition of some *At*PDF variants by actinonin. The enzyme concentration used in the assay was 100 nM. Prior to kinetic analysis for determination of *K*_I*app_ values, actinonin was incubated in the presence of each variant set at the final concentration for 10 min at 37°C; kinetic assay was started by adding a small volume of the substrate. For determination of *K*_I_, *k*_5_, and *k*_6_ values, actinonin was not pre-incubated with enzyme and kinetic assay was started by adding the enzyme.
(DOCX)

Movie S1 Dynamics of actinonin binding to peptide deformylase and closure of the active site.
(WMV)

Movie S2 Progressive motions of the main side chains at the active site and final locking of the hydrogen bond.
(WMV)

## Abbreviations

DSC: differential scanning calorimetry
*Fo*: formyl
PDF: peptide deformylase
r.m.s.d.: root mean square deviation
