# Supplementary material for: Trapping Conformational States Along Ligand-Binding Dynamics of Peptide Deformylase: The Impact of Induced Fit on Enzyme Catalysis
Source: PLoS Biol. 2011 May 24;9(5):e1001066. doi: 10.1371/journal.pbio.1001066 (PMC3101196; doi:10.1371/journal.pbio.1001066)
Supplement: Table S1 — Catalytic properties of AtPDF. Nm, not measurable; ND, not determined; WT, is wild-type. aKinetic constants were determined using the coupled assay as indicated in Materials and Methods with substrate Fo-Met-Ala-Ser, in the presence of 100 nM enzyme variant and 750 µM NiCl2, at 37°C. The relative value of k cat/K m for wild-type AtPDF was set at 100%. bData correspond to the binding constant of actinonin as obtained either from ITC or from enzymatic analysis when indicated with an asterisk. cData from Table S3. dGyration radii are from [83]. (DOC) [file pbio.1001066.s011.doc]

**Table S1. Catalytic properties of *At***PDF

| Enzyme  variant | *K*_m_ (mM) ^a^ | *k*_cat_ (sec^-1^)^a^ | *k*_cat_/*K*_m_  (mM^-1^ sec^-1^)^a^ | Relative  activity (%) | K_d_ (nM)^b^ | K_I*app_  (nM)^c^ | Gyration radius (Å)^d^ |
| --- | --- | --- | --- | --- | --- | --- | --- |
| **WT** | 6.5 ± 0.7 | 37 ± 2 | 5.7 ± 0.6 | **100** | 0.9*(4.5) | 2.3 ± 0.3 | 0.00 |
| **G41A** | 3.7 ± 0.4 | 4.3 ± 0.2 | 1.15 ± 0.2 | **20** | ND | ND | 0.77 |
| **G41Q** | 6.7 ± 1.7 | 0.14 ± 0.02 | 0.02 ± 0.01 | **0.35** | 48* | nm | 1.75 |
| **G41M** | 5.8 ± 1.5 | 0.39 ± 0.05 | 0.07 ± 0.01 | **1.2** | 32 | nm | 1.80 |
| **I42A** | 10.1 ± 2.6 | 23 ± 4 | 2.2 ± 0.2 | **39** | 3* | ND | 0.77 |
| **I42F** | 9.8 ± 2.0 | 24 ± 3 | 2.5 ± 0.2 | **44** | 2.8*(3.6) | 2.8 ± 0.2 | 1.90 |
| **I42N** | 5 ± 3 | 7 ± 3 | 0.9 ± 0.1 | **16** | 6.3* | 6.3 ± 0.4 | 1.45 |
| **I42W** | 5.3 ± 0.5 | 32 ± 1 | 5.9 ± 0.6 | **104** | 3.7 | 2.8 ± 0.4 | 2.21 |
| **I130A** | 5 ± 3 | 4 ± 2 | 0.6 ± 0.1 | **11** | ND | 1.5 ± 0.4 | 1.56 |
| **I130F** | 5.2 ± 1.0 | 4.1 ± 0.5 | 0.8 ± 0.1 | **14** | ND | ND | 1.90 |

nm not measurable, ND not determined, WT is wild-type.

^a^ kinetic constants were determined using the coupled assay as indicated in Materials and Methods with substrate *Fo*-Met-Ala-Ser, in the presence of 100 nM enzyme variant and 750 µM NiCl_2_, at 37°C. The relative value of *k*_cat_/*K*_m_ for wild-type *At*PDF was set at 100%.

^b^ data correspond to the binding constant of actinonin as obtained either from ITC or from enzymatic analysis when indicated with an asterisk.

^c^ data from **Table S3**.

^d^ gyration radii are from Ref.[83].
