# Supplementary material for: Trapping Conformational States Along Ligand-Binding Dynamics of Peptide Deformylase: The Impact of Induced Fit on Enzyme Catalysis
Source: PLoS Biol. 2011 May 24;9(5):e1001066. doi: 10.1371/journal.pbio.1001066 (PMC3101196; doi:10.1371/journal.pbio.1001066)
Supplement: Table S2 — Crystallographic data and refinement statistics. Values in parentheses are for the outer resolution shell. aRsym (I) = ΣhklΣi|Ihkl,i − |/ΣhklΣi|Ihkl,i|, where is the mean intensity of the multiple Ihkl,i observations for symmetry-related reflections. bRwork = 100× (Σhkl|Fobs − Fcalc|/Σhkl|Fobs|). Rfree is a test set including ∼5% of the data. cPercentage of residues in most-favored/additionally allowed/generously allowed/disallowed regions of the Ramachandran plot. dCompound 21 was added first, and actinonin afterwards. (DOC) [file pbio.1001066.s012.doc]

**Table S2**. Crystallographic data and refinement statistics

| Experimental conditions | wt  ---  (PEG3350) | wt  ---  (PEG550MME) | wt  actinonin  (PEG3350) | wt  6b  (PEG550MME) | wt  21  (PEG3350) | wt  21+ actinonin^d^  (PEG550MME) | G41Q  ---  (PEG3350) | G41Q  actinonin  (PEG3350) | G41M  ---  (PEG3350) | G41M  actinonin  (PEG3350) |
| --- | --- | --- | --- | --- | --- | --- | --- | --- | --- | --- |
| *Data* |  |  |  |  |  |  |  |  |  |  |
| Wavelength (Å) | 0.980 | 0.980 | 0.980 | 0.980 | 0.970 | 0.939 | 0.934 | 0.934 | 0.873 | 0.873 |
| Resolution (Å) | 2.0 | 2.0 | 2.0 | 3.0 | 1.3 | 1.9 | 2.3 | 2.4 | 2.1 | 2.4 |
| Space group | *P2_1_2_1_2_1_* | *P4_3_2_1_2* | *P2_1_2_1_2_1_* | *P4_3_2_1_2* | *P2_1_2_1_2_1_* | P*4*_3_*2*_1_*2* | P*4*_3_*2*_1_*2* | *P4_3_2_1_2* | *P4_3_2_1_2* | *P2_1_2_1_2_1_* |
| Number of molecules per asymmetric unit | 2 | 1 | 2 | 1 | 2 | 1 | 1 | 1 | 2 | 4 |
| Unit cell parameters (Å) | a=55.97  b=55.94  c=148.55 | a=56.32  b=56.32  c=148.44 | a=56.56  b=56.47  c=145.95 | a=57.50  b=57.50  c=144.84 | a=55.60  b=55.61  c=149.53 | a=56.57  b=56.57  c=146.11 | a=56.93  b=56.93  c=147.41 | a=56.63  b=56.63  c=146.55 | a=66.28  b=66.28  c=194.29 | a=66.64  b=66.73  c=189.69 |
| Completeness (%) | 99.0 (97.3) | 98.8 (98.9) | 98.9 (93.2) | 98.1 (98.1) | 98.5 (96.9) | 99.7 (99.8) | 98.3 (94.0) | 98.8 (93.5) | 99.7 (98.2) | 97.5 (96.1) |
| *I/σ* | 14.48 (5.06) | 12.60 (2.65) | 13.57 (4.99) | 13.50 (3.00) | 19.33 (5.10) | 18.95 (4.14) | 136.64 (5.82) | 22.77 (6.10) | 21.56 (6.80) | 21.28 (4.69) |
| *R*_sym_ (%) ^a^ | 9.4 (45.5) | 5.0 (40.9) | 10.1 (38.8) | 4.6 (24.1) | 6.6 (39.8) | 5.9 (33.5) | 11.6 (30.3) | 8.7 (42.6) | 8.4 (37.4) | 5.7 (42.0) |
| *Refinement* |  |  |  |  |  |  |  |  |  |  |
| Number of ligand molecules | 11 Zn^2+^  494 H_2_0  - | 6 Zn^2+^  251 H_2_0  - | 9 Zn^2+^  435 H_2_0  2 actinonin | 6 Zn^2+^  84 H_2_O  1 ***6b*** | 15 Zn^2+^  620 H_2_0  2 ***21*** | 5 Zn^2+^  251 H_2_0  1 actinonin | 6 Zn^2+^  280 H_2_0  - | 5 Zn^2+^  149 H_2_O  1actinonin | 7 Zn^2+^  396 H_2_0  - | 19 Zn^2+^  464 H_2_0  4 actinonin |
| *R*_work_ / *R*_free_ (%) | 21.70 / 28.38 | 20.26 / 25.75 | 21.48 / 26.45 | 20.04 / 28.51 | 15.95 / 16.95 | 18.18 / 20.99 | 25.73 / 35.89 | 22.87 / 30.85 | 17.10 / 21.22 | 22.62 / 29.34 |
| rmsd of Bonds (Å) / Angles (deg) | 0.022 / 2.036 | 0.024 / 1.879 | 0.022 / 1.964 | 0.050 / 3.900 | 0.006 / 1.143 | 0.015 / 1.387 | 0.033 / 2.935 | 0.029 / 2.294 | 0.017 / 1.532 | 0.0077 / 1.279 |
| Ramachandran plot ^c^ | 91.3/8.1/0.6/ 0.0 | 91.6/7.7/0.6/ 0.0 | 92.6/6.8/0.6/ 0.0 | 79.1/20.3/0.7/0.0 | 91.0/9.0/0.0 / 0.0 | 91.7/7.7/0.6 / 0.0 | 83.4/15.3/1.3/0.0 | 88.8/11.5/0.0/0.0 | 91.5/7.9/0.6/0.0 | 87.8/11.7/0.5/0.0 |
| *PDB accession code* | 3M6O | 3PN2 | 3M6P | 3O3J | 3PN3 | 3PN4 | 3PN5 | 3M6Q | 3PN6 | 3M6R |

Values in parenthesis are for the outer resolution shell.

^a^ R_sym_ (I) = Σ*_hkl_Σ_i_*|*I_hkl,i_* - <*I_hkl_*>|/Σ*_hkl_Σi*|*I_hkl,i_*|, where <*I_hkl_*> is the mean intensity of the multiple *I_hkl,i_* observations for symmetry-related reflections.

^b^ R_work_ = 100 x (Σ*_hkl_*|Fobs – Fcalc|/Σ _hkl_|Fobs|). R_free_ is a test set including ~5% of the data.

^c^ Percentage of residues in most-favored/additionally allowed/generously allowed/disallowed regions of the Ramachandran plot.

^d^ Compound ***21*** was added first, and actinonin afterwards.
