# Supplementary material for: Trapping Conformational States Along Ligand-Binding Dynamics of Peptide Deformylase: The Impact of Induced Fit on Enzyme Catalysis
Source: PLoS Biol. 2011 May 24;9(5):e1001066. doi: 10.1371/journal.pbio.1001066 (PMC3101196; doi:10.1371/journal.pbio.1001066)
Supplement: Table S3 — Kinetic parameters for inhibition of some AtPDF variants by actinonin. The enzyme concentration used in the assay was 100 nM. Prior to kinetic analysis for determination of K I*app values, actinonin was incubated in the presence of each variant set at the final concentration for 10 min at 37°C; kinetic assay was started by adding a small volume of the substrate. For determination of K I, k 5, and k 6 values, actinonin was not pre-incubated with enzyme and kinetic assay was started by adding the enzyme. (DOCX) [file pbio.1001066.s013.docx]

**Table S3. Kinetic parameters for inhibition of some *At*PDF variants by actinonin**

|  | Wild-type | I42W | I42F | I42N | I130A |
| --- | --- | --- | --- | --- | --- |
| *K*_I_ (nM) | 140 ± 10 | 97 ± 6 | 43 ± 5 | 39 ± 4 | 36 ± 5 |
| *K*_I*app_ (nM) | 2.3 ± 0.3 | 2.8 ± 0.4 | 2.8 ± 0.2 | 6.3 ± 0.4 | 1.5 ± 0.4 |
| *K*_I_/*K*_I*app_ | 61 ± 12 | 35 ± 7 | 15 ± 3 | 6 ± 2 | 24 ± 5 |
| *k*_5_ (s^-1^) x 10^3^ | 63 ± 6 | 52 ± 5 | 89 ± 9 | 52 ± 5 | 49 ± 5 |
| *k*_6_ (s^-1^) x 10^4^ | 10 ± 1 | 16 ± 2 | 62 ± 6 | 101 ± 10 | 21 ± 2 |

The enzyme concentration used in the assay was 100 nM. Prior to kinetic analysis for determination of *K*_I*app_ values, actinonin was incubated in the presence of each variant set at the final concentration for ten minutes at 37°C; kinetic assay was started by adding a small volume of the substrate. For determination of *K*_I_, *k*_5_ and *k*_6_ values, actinonin was not pre-incubated with enzyme and kinetic assay was started by adding the enzyme.
